# Dynamic Path Planning for Forklift AGV Based on Smoothing A* and Improved DWA Hybrid Algorithm

**DOI:** 10.3390/s22187079

**Published:** 2022-09-19

**Authors:** Bin Wu, Xiaonan Chi, Congcong Zhao, Wei Zhang, Yi Lu, Di Jiang

**Affiliations:** College of Mechanical and Electronic Engineering, Nanjing Forestry University, Nanjing 210037, China

**Keywords:** forklift AGV, path planning, hybrid algorithms, improved A* algorithm, improved DWA

## Abstract

FAGV is a kind of heavy equipment in the storage environment. Its path needs to be simple and smooth and should be able to avoid sudden obstacles in the process of driving. According to the environmental characteristics of intelligent storage and the task requirements of FAGV, this paper proposed a hybrid dynamic path planning algorithm for FAGV based on improved A* and improved DWA. The improved A* algorithm can plan the global optimal path more suitable for FAGV. The improved evaluation function of DWA can ensure that the local path of FAGV is closer to the global path. DWA combines the rolling window method for local path planning to avoid sudden unknown static and dynamic obstacles. In addition, this paper verifies the effectiveness of the algorithm through simulation. The simulation results show that the algorithm can avoid obstacles dynamically without being far away from the global optimal path.

## 1. Introduction

**Figure 1:**
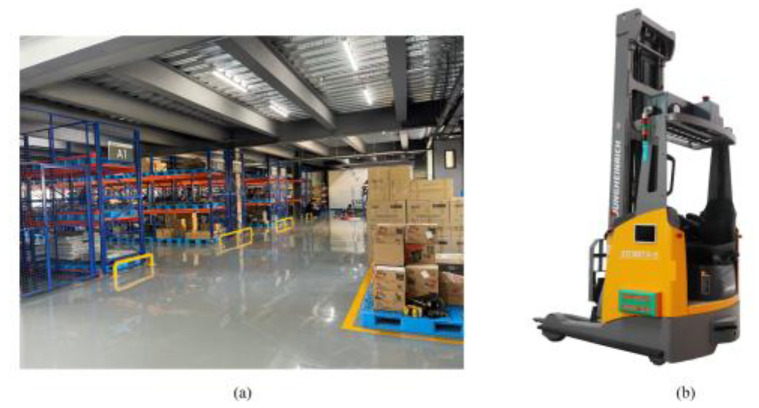
(**a**) FAGV working environment diagram, (**b**) forward-moving FAGV [6].

Forklift Automated Guided Vehicle (FAGV) is a kind of transport vehicle with a rechargeable battery as the energy source. Under the guidance of automatic navigation and positioning devices such as navigation laser scanners and binocular cameras, it can run according to the desired path planned by the guiding system. FAGV has the functions of autonomous obstacle avoidance and fault alarm, and can also carry out various transfer functions. As an important part of AGV, FAGV plays an irreplaceable role in heavy load, special handling, and other scenes. Figure 1a shows the working environment of FAGV, and Figure 1b is a photograph of a FAGV. AGV is currently widely used in warehouses [1], ports [2], and factories [3]. In recent years, forklift unmanned transformation has become a trend. In the storage environment, the main tasks of FAGV include road scene recognition, path planning, tracking control [4], and local obstacle avoidance [5]. Among them, path planning and local obstacle avoidance are the key points in the research of forklift unmanned transformation.

Path planning refers to finding collision-free handling paths that meet the requirements of AGV operation according to the rules set in advance (with the least time or the shortest distance) when AGV carries out cargo handling tasks [7]. Path planning includes the following three aspects [8]: (1) starting point and target point; (2) bypassing the known static obstacles; (3) the best path according to the set rules. Due to the large size and weight of FAGV, the path of FAGV needs to be smoother than ordinary AGV. Moreover, there are often unknown static or dynamic obstacles in the storage environment, such as the running FAGV or walking people. FAGV usually carries heavy goods. If they cannot avoid obstacles in time, it will cause serious consequences. Therefore, the path planning of FAGV should not only have shorter distance and less time but also avoid obstacles in time and safety. According to these characteristics of FAGV, this paper studies the path planning algorithm that meets the requirements of FAGV.

According to the mastery of warehouse map information, FAGV path planning can be divided into local dynamic path planning and global path planning [9]. Global path planning is to plan a barrier-free shortest path [10] for FAGV with known warehouse map information [11]. The global path search methods mainly include heuristic search algorithms, sampling search algorithms, and intelligent algorithms. The heuristic search algorithm includes the Dijkstra algorithm [12] and BFS algorithm [13], etc. The Dijkstra algorithm can find the shortest path, but the efficiency is relatively lower [14]. The BFS algorithm uses a heuristic function to search and the efficiency is high, but the planned path is not the shortest. The A* algorithm [15] is composed of the best-first search algorithm (BFS) and the Dijkstra algorithm, so it not only has the search speed of the BFS algorithm (using a heuristic function to guide itself to quickly guide the target node) but also can make the search path the shortest like the Dijkstra algorithm. Sampling search algorithm including fast extended random tree (RRT) [16] and its optimization algorithm [17]. This algorithm takes the initial position as the root node of the random tree and randomly points out the growth direction of the tree on the map. When the target node is included in the sub-node of the tree, the search ends. This method is fast and can search in multidimensional space. Chi W Z et al. [18] proposed a heuristic path planning algorithm based on a generalized Voronoi diagram (GVD) to generate heuristic paths, guide the sampling process of RRTs, and further improve the efficiency of motion planning of RRTs. Jiang et al. [19] proposed an improved bidirectional A* search algorithm from the perspective of heuristic function and search direction. In the heuristic function of the A* algorithm, the chord factor was introduced to optimize the direction of the path search. The unidirectional search from the starting point to the endpoint was changed to a bidirectional simultaneous search, which improved the problems of long planning paths and low unidirectional search efficiency in the path planning of electric disinfection vehicles. Tang G et al. [20] proposed a geometric A* algorithm for AGV global path planning in a port environment, which reduced the number of path nodes and the number of turns. Intelligent algorithms include particle swarm optimization (PSO), genetic algorithms, ant colony algorithms, simulated annealing algorithms, etc. Intelligent algorithms such as the PSO algorithm for path planning have the characteristics of easy implementation, high precision, and fast convergence speed, but there are problems such as easy to fall into local optimum and long planning path [21]. Xu L et al. [22] proposed a smooth path planning method for mobile robots based on the quartic Bezier transition curve and an improved PSO algorithm. By analyzing the stability of the algorithm, the parameter relationship to ensure the convergence of the proposed adaptive weighted delay speed PSO algorithm is derived. Dang et al. [23] proposed a chain navigation grid for a virtual reality large-scale crowd evacuation simulation. However, the above separate global planning algorithms cannot be used in unknown dynamic environments, and are not suitable for heavy equipment such as FAGV. Therefore, it is necessary to optimize the global planning algorithm and combine it with local planning.

Local path planning is to detect the surrounding environment of FAGV by means of a safety laser scanner, motion sensor, and other tools to obtain the location information and motion of unknown static and dynamic obstacles around and find a path that can bypass obstacles. The local path planning algorithm integrates environmental modeling and path searching and has good anti-interference ability to environmental error and noise, which can provide real-time feedback and correction for planning results. Local path planning algorithms mainly include classical algorithms such as dynamic window algorithms [24], artificial potential field algorithms [25], and intelligent algorithms such as neural network algorithms and reinforcement learning algorithms. In order to further improve the path planning of mobile robots in complex dynamic environments, Liwei Yang et al. [26] proposed an improved hybrid algorithm combining the excellent searchability of the ant colony algorithm (ACO) for global path and the advantages of the dynamic window method (DWA) for local obstacle avoidance. Sollehudin I. M et al. [27] introduced the artificial potential field method into the electric wheelchair control system to help the electric wheelchair complete its daily work. Lin, Zenan, et al. [28] proposed an improved artificial potential field model, using a sub-objective strategy to solve a local minimum problem. In order to show the adaptive selection characteristics of robot sub-goals, the obstacle potential field function is established, and the effectiveness of adaptive characteristics is verified by path planning simulation. Vahide Bulut [29] proposed an improved ε-greed epsilon-greed Q-learning (IEGQL) algorithm and proposed a new reward function and mathematical model to ensure that mobile robots can obtain environmental knowledge in advance and provide optimal selection while ensuring rapid convergence. Simple local path planning cannot complete the task of FAGV, so it is necessary to optimize the local planning algorithm and mix the global planning algorithm.

An ideal dynamic path planning algorithm for FAGV should not only be able to plan a path with a short length and less time—which meets the dynamics requirements of FAGV, namely a simple and smooth path—but also avoid static and dynamic obstacles in the process of driving. In order to solve the above problems, this paper proposes a hybrid dynamic path planning algorithm for FAGV based on improved A* and improved DWA. The contribution of the proposed algorithm to the FAGV path planning problem is as follows:

1. Aiming at the problem of redundant path points and multiple turning points in the planning path of the traditional A* algorithm. This paper improves the A* algorithm in path smoothing.
2. When the dynamic window method is used to avoid obstacles, the local path may be far away from the global optimal path. And the excessive speed of the forklift may cause accidents when it is close to the obstacle. To solve the above problems, this paper introduces two evaluation indexes in the trajectory evaluation function: the distance between the local path and the global path and the distance between the trajectory point and the local sub-target point, which can make the local path closer to the global optimal path, and reduce the speed of the FAGV approaching the local sub-target point, and avoid the FAGV crossing the target point or oscillation due to the excessive speed. The FAGV uses the rolling window method for collision prediction in the process of moving and then calls the improved DWA for local path planning and safe avoidance of obstacles to return to the global optimal path in time.

## 2. Global Path Planning Based on Improved A * Algorithm

### 2.1. Traditional A* Algorithm

The A* algorithm [15] uses the path length evaluation function *f*(*n*) to evaluate the path length. The basic idea is to sort the cost of the optional nodes around the current node, select the least-cost node, and repeat the cycle until it extends to the target point. The formula is as follows:

(1) $$\begin{array}{c} f\left(n\right)=g\left(n\right)+h\left(n\right) \end{array}$$

In the formula, *g*(*n*) is the actual cost from the starting point of the FAGV path to the current node n, and *h*(*n*) is the minimum estimation cost from node n to the target endpoint. If *h*(*n*) is zero, then only *g*(*n*) works and the A* algorithm degenerates into the Dijkstra algorithm; if *h*(*n*) is much larger than *g*(*n*), then *g*(*n*) can be approximately regarded as zero, and the A* algorithm degenerates into the BFS algorithm. *h*(*n*) can be selected according to the actual working environment, *h*(*n*) selection should meet a requirement that is not higher than the actual minimum cost of node n to the endpoint.

### 2.2. Improved A* Algorithm

In the known obstacle space, the A* algorithm can avoid obstacles correctly, find the shortest path and complete the global path planning requirements in the initial stage. However, the number of path turns is relatively large, and the smoothness is relatively poor. The traditional A* algorithm is not suitable for large transport equipment such as FAGV. To solve this problem, this paper proposes an improved A* algorithm that can remove redundant nodes and reduce the turning times.

**Figure 2:**
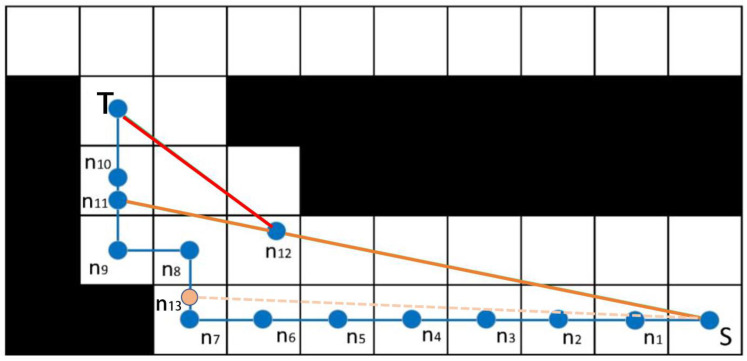
Raster path map (the starting point is S, and the target point is T).

As shown in Figure 2, the path planned by the A* algorithm is (S, $n_{1}$, $n_{2}$, $n_{3}$, $n_{4}$, $n_{5}$, $n_{6}$, $n_{7}$, $n_{8}$, $n_{9}$, $n_{10}$, T), which has multiple turns and poor smoothness. To solve the above problems, this paper optimizes the smoothness of the A* algorithm. The specific optimization steps are as follows:

Step 1: If the connection distance between non-adjacent nodes is less than the planned connection path distance and the connection does not collide with the obstacle, then the intermediate node belongs to redundant nodes, which can be deleted. Therefore, other nodes outside the first and last two nodes in the same direction are redundant nodes, which should be deleted. Only the initial node, the target node, and the middle inflection point are saved, and the reserved path is (S, $n_{7}$, $n_{8}$, $n_{9}$, T).

Step 2: Starting from the starting point S, a node is taken every certain step between the reserved two inflection points $n_{i}$, $n_{j}$, such as $n_{13}$, and the selected node is connected to the previous path node to check whether there is an obstacle between the two points. If there is no obstacle, the current node is selected as a new path node. If there is an obstacle, the node is abandoned, and the reserved path is (S, $n_{11}$, T).

Step 3: Change the optimization direction and retake the point from the target point to the starting point step by step. The remaining path is (S, $n_{12}$, T). Output optimization path, path optimization end.

## 3. Local Path Planning Based on Improved DWA

Under the global map with complete information about the environment and obstacles, the A* algorithm can conduct navigation well. However, there are some emergencies in the actual situation, such as unexpected obstacles or walking people on the original path. If appropriate measures are not taken, FAGV collides easily with obstacles. Therefore, to realize the real-time obstacle avoidance of the robot, this paper uses the DWA algorithm with local obstacle avoidance ability to plan the local path and avoid obstacles to ensure the safety of FAGV.

### 3.1. Basic Principle of DWA

The dynamic window algorithm mainly samples a variety of speeds in the speed space (including linear velocity and angular velocity) and uses this group of speeds to simulate the motion trajectory of FAGV within a certain time. After obtaining multiple sets of corresponding trajectories of speed, it uses certain evaluation rules to evaluate them and selects the corresponding speed of the optimal trajectory to drive the FAGV forward.

### 3.2. Kinetic Model of FAGV

**Figure 3:**
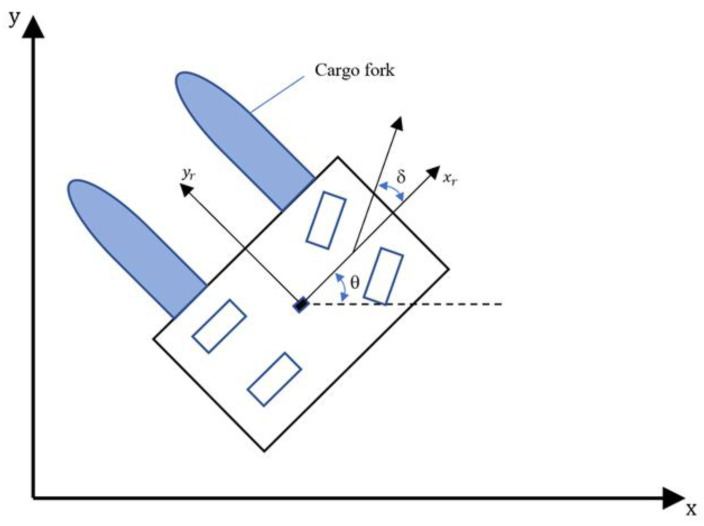
Kinetic model diagram of FAGV.

The premise of using the dynamic window method to simulate the motion trajectory is to know the kinetic model of FAGV. In this paper, the discrete kinematic model of the Ackerman steering vehicle [30] is used as the kinetic model of FAGV, and the relationship between vehicle pose (*x*, *y*, *θ*) and velocity (*v*, *ω*) can be obtained. Assuming that the trajectory is a circular arc, when the rotation speed is 0, the circular arc is approximately a straight line, and a pair of linear velocity and angular velocity (*v*, *ω*) represents a circular arc trajectory. When calculating the moving trajectory of FAGV at adjacent moments, the moving trajectory at adjacent moments can be approximated as a straight line due to the short time interval and moving distance. Since the FAGV does not move in all directions, it can only move forward and rotate, and cannot move longitudinally, thus the distance of the FAGV moving along the $y_{r}$ axis of its own coordinate system is not considered when calculating the trajectory of the FAGV. Assuming that the FAGV moves *v*∙∆*t* along the $x_{r}$ axis of its own coordinate system, the distance is projected on the x-axis and y-axis of the world coordinate system, as shown in Figure 3, and the coordinate increments ∆*x* and ∆*y* of the FAGV in the world coordinate system at the next moment relative to the previous moment can be obtained:

(2) $$\begin{array}{c} \{\begin{array}{c} ∆x=v\;\cdot ∆t\;\cdot \cos(\theta_{t})\\ ∆y=v\;\cdot ∆t\;\cdot \sin(\theta_{t}) \end{array} \end{array}$$

In order to calculate the trajectory of FAGV in a period of time, the displacement increment in this period can be accumulated:

(3) $$\begin{array}{c} \{\begin{array}{c} x=x_{0}+v∆t\cos(\theta_{t})\\ y=y_{0}+v∆t\sin(\theta_{t})\\ \theta_{t}=\theta_{t}+\omega ∆t \end{array} \end{array}$$

### 3.3. The Optimized Trajectory Evaluation Function

In the traditional dynamic window approach, the indicators of the evaluation function are azimuth, void, and velocity. Its evaluation function *G*(*v*, *ω*) [31] is:

(4) $$\begin{array}{c} G\left(v,\omega \right)=\sigma \left(\alpha \cdot \mathrm{heading}\left(v,\omega \right)+\beta \cdot \mathrm{vel}\left(v,\omega \right)+\gamma \cdot \mathrm{dist}\left(v,\omega \right)\right) \end{array}$$

In the formula, heading(v,ω) is the direction angle evaluation index, dist(v,ω) is the distance between the current trajectory and the nearest obstacle, and vel(v,ω) is the current speed evaluation function. *α*, *β*, and *γ* are the weighting coefficients of the three evaluations. To avoid one of the three values being too large and too dominant, the evaluation function is obtained by multiplying and adding the corresponding coefficients after smoothing. Finally, the smoothing factor σ is used to normalize the objective function.

The main purpose of this paper is to avoid obstacles by using the DWA for local path planning. The local path planned by DWA usually deviates greatly from the global path when avoiding obstacles, which not only causes the global optimality of the path to deteriorate but also makes FAGV walk many useless paths, resulting in a waste of time and energy. Therefore, the distance from the reference trajectory to the global path is considered as an evaluation index to make the obstacle avoidance path as close as possible to the global optimal path, so as to improve the global optimality of the dynamic window method.

At the same time, the distance between the reference trajectory point and the local sub-target point is added as the evaluation index, which can reduce the speed of FAGV approaching the local sub-target point and avoid the phenomenon of FAGV crossing the target point or shaking due to the excessive speed.

In summary, in order to improve the global and local optimality of the dynamic window method, a new evaluation function is designed in this paper. The distance between the reference trajectory and the global path dist − 1(*v*, *ω*) and the distance between the reference trajectory point and the local sub-target point dist − 2(*v*, *ω*) is added to the evaluation index. The trajectory with the lowest cost of the evaluation function *G*(*v*, *ω*) is the optimal trajectory, and the evaluation function can be rewritten as:

(5) $$\begin{array}{l} G\left(v,\omega \right)=\sigma \left(\begin{array}{l} \alpha \cdot \mathrm{heading}\left(v,\omega \right)+\beta \times \mathrm{vel}\left(v,\omega \right)+\gamma \cdot \mathrm{dist}\left(v,\omega \right)+\\ \eta \cdot \mathrm{dist}-1\left(v,\omega \right)+\lambda \cdot \mathrm{dist}-2\left(v,\omega \right) \end{array}\right) \end{array}$$

## 4. Collision Prediction Based on Rolling Window

### 4.1. Local Collision Prediction

**Figure 4:**
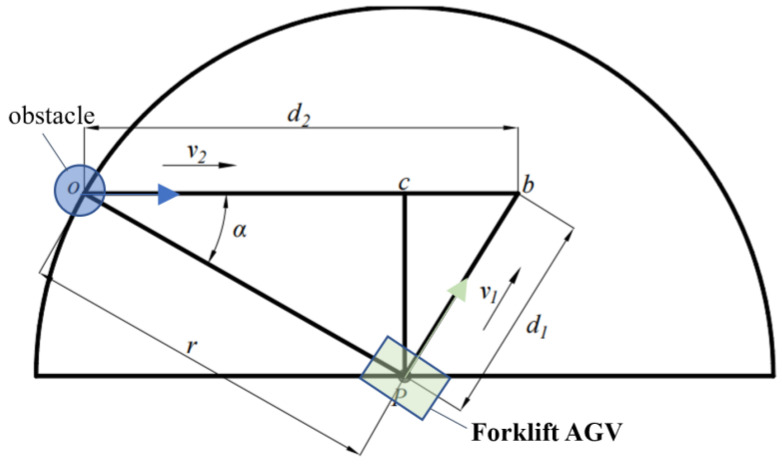
Model observation map.

Before the local planning, it is necessary to predict the local collision to determine whether the FAGV will collide with the obstacle. If the collision is going to occur, the corresponding collision avoidance strategy will be taken according to the type of obstacle. In this section, the obstacle collision is predicted based on the rolling window method [32], and the corresponding obstacle avoidance measures are formulated according to different collision situations. The rolling window method takes the moving FAGV as the center and the detection range of the laser radar as the radius to construct the rolling window (Figure 4). According to the environmental information collected by the laser radar, the environmental information in the window is updated to analyze and predict the trajectory of the obstacle and plan the local path.

If the FAGV detects static obstacles on the road, the improved dynamic window algorithm is used for local path planning before the safe distance from static obstacles.

If the FAGV detects the dynamic obstacle on the road, the collision prediction is carried out to calculate whether the shortest distance between the FAGV and the dynamic obstacle is less than the safe distance. If it is less than, the collision will occur, and if it is greater, the collision will not occur.

The detection range of lidar is a fan-shaped region with its own center and radius r. The running speed of FAGV is $v_{1}$, the cycle is T, the step size is *s*, the direction is $\theta_{1}$, and the position is $p_{1r}$; the moving speed of dynamic obstacle o is $v_{2}$, the direction is $\theta_{2}$, and the position is $p_{2o}$; according to obstacle avoidance requirements:

(6) $$\begin{array}{c} \left(v_{1}+v_{2}\right)\cdot T<r \end{array}$$

T *= s/v*, then:

(7) $$\begin{array}{c} v_{2}<\left(\frac{r}{s}-1\right)\cdot v_{1} \end{array}$$

Figure 4 is the model observation diagram. According to the motion relationship between the FAGV and the obstacle, the collision point between the forklift AGV and the dynamic obstacle is calculated.

Knowing from the motion relationship of an object that:

(8) d1v1=d2v2

And:

(9) $$\begin{array}{l} \{\begin{array}{l} d_{0}=pb=\sqrt{pc^{2}+bc^{2}}\\ bc=\left|d_{2}-r\cdot \cos\left(\alpha \right)\right|\\ pc=r\cdot \sin\left(\alpha \right) \end{array} \end{array}$$

Simultaneous (8) and (9):

(10) $$\begin{array}{c} \left(v_{2}^{2}-v_{1}^{2}\right)\cdot d_{1}^{2}-2rd_{2}v_{2}^{2}\cos\left(\alpha \right)+r^{2}v_{2}^{2}=0 \end{array}$$

Solution of *d_2_* is:

(11) $$\begin{array}{l} \{\begin{array}{l} d_{2}=\frac{2rv_{2}^{2}\cos\left(\alpha \right)\pm \sqrt{\Delta }}{2\left(v_{2}^{2}-v_{1}^{2}\right)}\\ \Delta =4r^{2}v_{2}^{2}\left(v_{1}^{2}-v_{2}^{2}\sin^{2}\left(\alpha \right)\right) \end{array} \end{array}$$

If ∆ ≥ 0, indicating that the FAGV will collide with the dynamic obstacle, $d_{2}$ is the distance of the dynamic obstacle movement.

Assuming that the current speed direction of FAGV is constant, the speed direction of dynamic obstacles is also constant. According to the current rolling window, the position and orientation of the FAGV and the dynamic obstacle $p_{2r}$ and $p_{2o}$ are predicted at the next moment.

(12) $$\begin{array}{c} \{\begin{array}{c} p_{2r}=p_{1r}+T\cdot v_{1}\cdot \frac{\theta_{1}}{\mathrm{norm}\left(\theta_{1}\right)}\\ p_{2o}=p_{1o}+T\cdot v_{2}\cdot \frac{\theta_{2}}{\mathrm{norm}\left(\theta_{2}\right)} \end{array} \end{array}$$

In the Formula, $\mathrm{norm}\left(\theta_{1}\right)$ and $\mathrm{norm}\left(\theta_{2}\right)$ are two-norms of $\theta_{1}$ and $\theta_{2}$. According to Formulas (11) and (12), the collision point position between FAGV and dynamic obstacles can be obtained.

### 4.2. Selection of Local Sub-Target Points

In the local path planning algorithm based on the rolling window method, the most important thing is to select the local sub-goal points of the current window. The rolling window at time t is denoted as *W (t)*. The selection idea of the local sub-goal point $p_{1}$ is that if the global goal point $p_{2}$ is located in the current rolling window, the local sub-goal point of the current window is $p_{2}$. If the global target point $p_{2}$ is not in the current scroll window, select the window boundary point p which minimizes *f(p)* as the local sub-target point. After selecting the local sub-target, the current speed direction of the FAGV is changed, and the dynamic window algorithm is used for local path planning to control the FAGV to the local sub-target point, so as to achieve the purpose of obstacle avoidance. The selection formula of local sub-target points is as follows:

(13) $$\begin{array}{l} p_{1}=\{\begin{array}{l} p_{2},p_{2}\in \partial w\left(t\right)\\ minf\left(p\right),p\in \partial w\left(t\right),p_{2}\notin \partial w\left(t\right) \end{array} \end{array}$$

**Figure 5:**
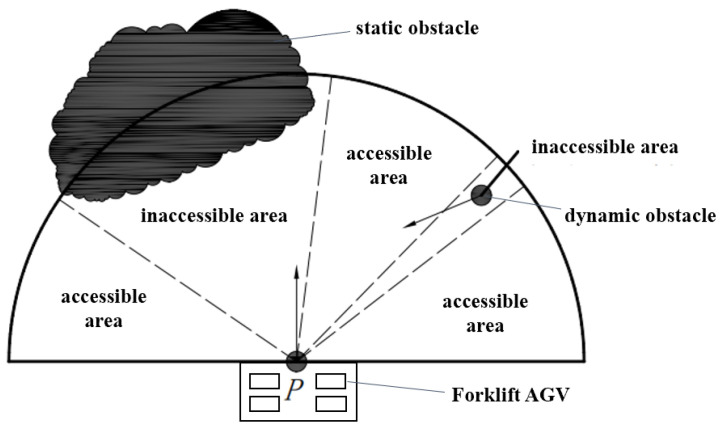
Reachable area division.

In the formula, *f(p)* draws on the idea of the heuristic function of the A* algorithm. The formula of *f(p)* is f(p)=g(p)+h(p). *g*(*p*) is the cost from the current position to *p*, and *h*(*p*) is the cost from p to the end. *gip*) is determined by whether p is in the reachable region. If the window boundary point p is in the reachable region, then *g*(*p*) = 0. If the window boundary point p is not in the reachable region, *g*(*p*) = +∞, the reachable region is divided as shown in Figure 5. Since the information outside the rolling window of FAGV is unknown, *h*(*p*) is estimated by the Euclidean distance from the point $p_{1}$ to the global target point $p_{2}$. This method of selecting local target points can fully cope with the sudden static and dynamic obstacles on the route of FAGV, and ensure the FAGV avoids obstacles smoothly.

### 4.3. Collision Avoidance Strategy

Now, we analyze the collisions that can be encountered in the warehouse environment and give the corresponding strategies.

1. **Static obstacles in front:** Call the improved DWA for local path planning to avoid obstacles.
2. **Dynamic obstacles coming opposite:** Make collision prediction and calculate collision location, then call DWA for local path planning.
3. **Obstacles on the front side:** Predict whether a collision occurs and if so, call DWA to avoid obstacles; if not, continue along the global path.

## 5. Dynamic Path Planning Algorithm of FAGV Based on Improved A* and Improved DWA Hybrid

**Figure 6:**
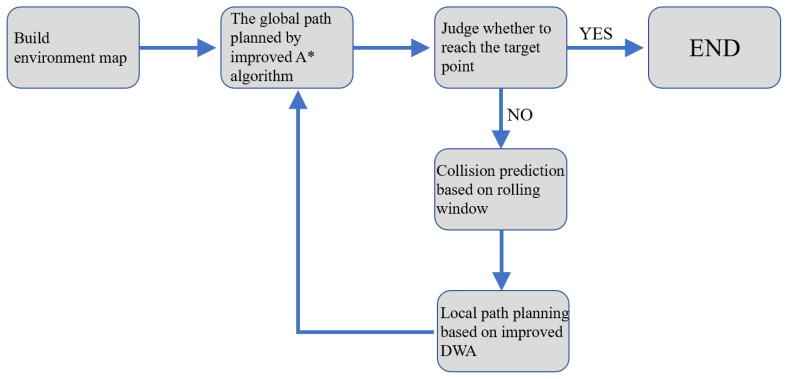
Process diagram of hybrid algorithm.

By improving the traditional A* and DWA algorithm, the dynamic path planning algorithm for FAGV can be concluded as follows. In the process of driving along the global shortest path planned by the improved A* algorithm, the FAGV constantly updates the map information in the rolling window and makes collision predictions. Improved DWA is called for local path planning if there are unknown obstacles in front of the collision. After avoiding obstacles, the FAGV returns to the global path to continue driving. The process of the hybrid algorithm is shown in Figure 6.

The algorithm process is as follows:

Step 1: Build an environment map, and set the starting point and end point of forklift AGV operation.

Step 2: Using an improved A* algorithm for global path planning, find the shortest barrier-free path.

Step 3: Determine whether the FAGV is exercised to the target point and if so, end the algorithm, otherwise execute step 4.

Step 4: Use laser radar and other sensors carried by FAGV to collect environmental information around the body, update the internal environment of the rolling window, and determine whether there are unknown static and dynamic obstacles.

Step 5: If there is no obstacle, continue to follow the global path, if there are obstacles, collision prediction, according to the corresponding results to take different obstacle avoidance measures.

Step 6: Use the improved dynamic window algorithm for local path planning to avoid obstacles. FAGV travels along the local path and returns to the global path after obstacle avoidance is completed. Return to step 3.

**Figure 7:**
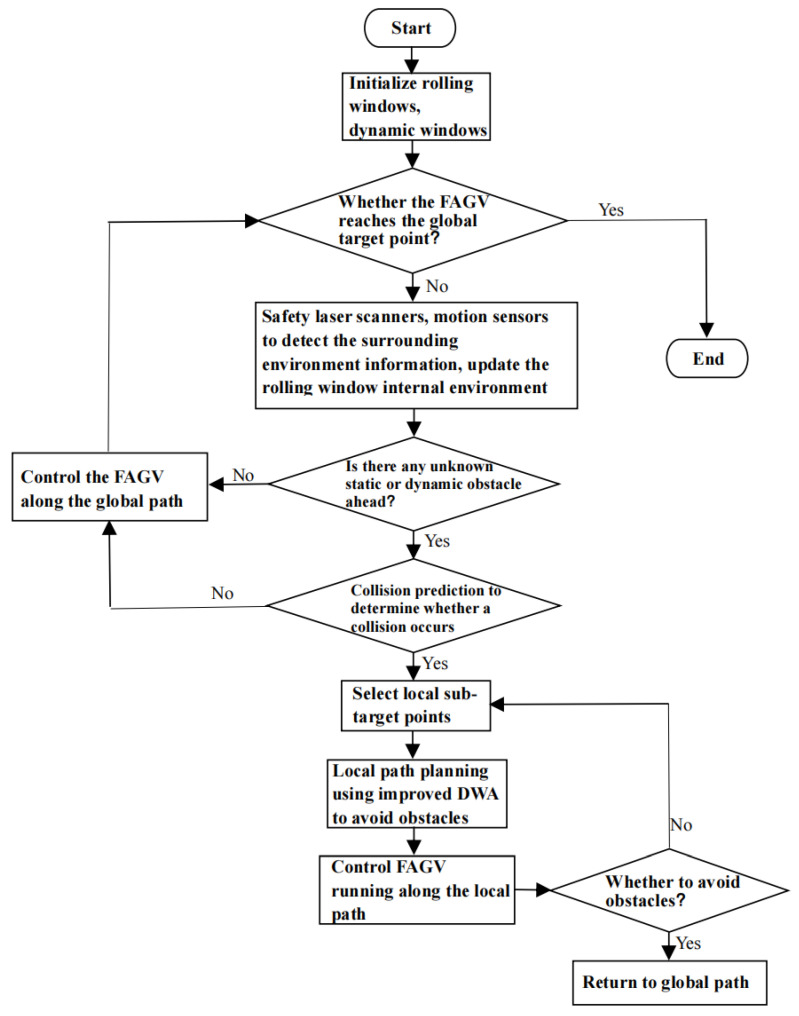
Flow chart of the FAGV dynamic path planning hybrid algorithm.

The flow chart of the FAGV dynamic path planning hybrid algorithm based on improved A* and DWA is shown in Figure 7.

## 6. Simulation Analysis

### 6.1. Simulation Results of Global and Local Path Planning

**Figure 8:**
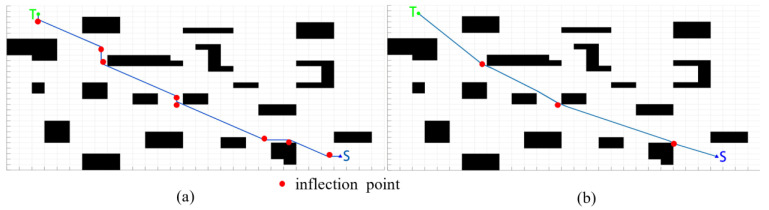
Algorithm simulation results: (**a**) traditional A* (**b**) improved A*.

Firstly, we simulate the global path planning. As shown in Figure 8, black blocks represent known obstacles and blank areas represent movable areas. The starting point is S, and the target point is T. The number of inflection points of the path can be regarded as the number of turns. Compared with the A* algorithm and improved A* algorithm, in the same map, the path planned by the traditional A* algorithm turns eight times in total, while the path planned by the improved A * algorithm only turns three times, and the turning times of the improved path are reduced by 62.5%. Moreover, the improved A* planning has a smaller turning angle and higher smoothness, which is more in line with the dynamic characteristics of FAGV.

In order to verify the effectiveness of the new indicators of the trajectory evaluation function, the improved DWA is simulated (Figure 7 and Figure 8). Set the traditional evaluation function parameters α = 0.2, β = 0.3, γ = 0.5. The evaluation function parameters designed in this paper are α = 0.2, β = 0.3, γ = 0.15, η = 0.2, λ = 0.15. Speed parameter settings are shown in Table 1.

**Figure 9:**
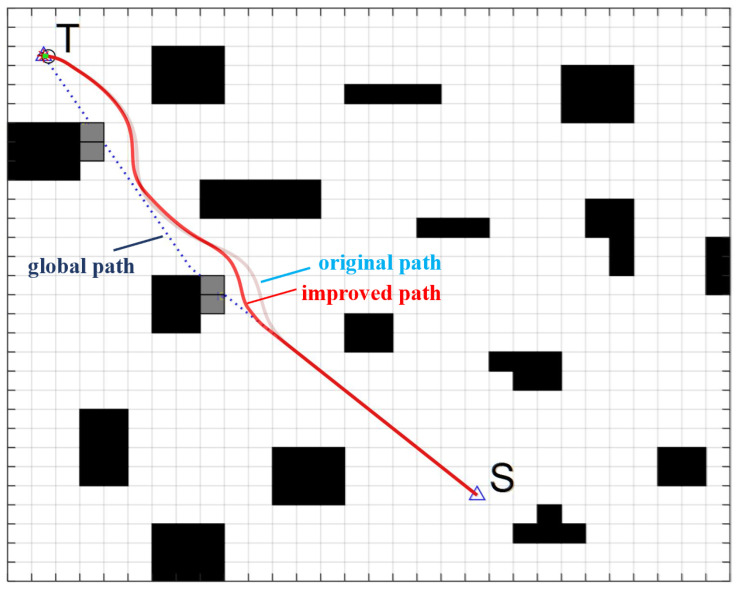
Comparison of local paths before and after improvement of the evaluation function.

**Figure 10:**
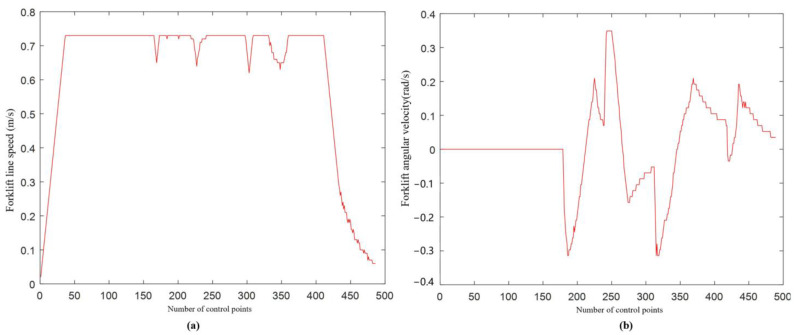
Simulation results of original evaluation function: (**a**) linear speed of FAGV (**b**) angular velocity of FAGV.

**Figure 11:**
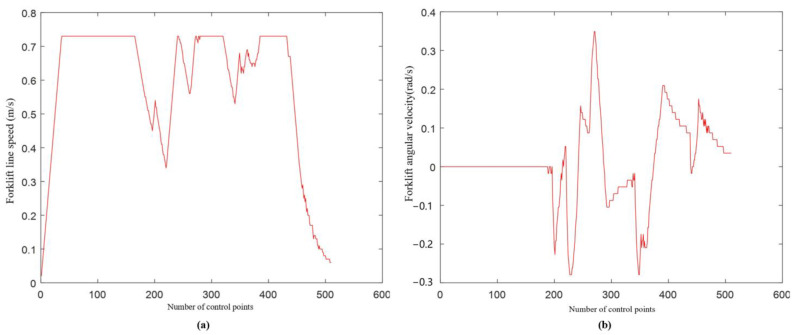
Simulation results of improved evaluation function: (**a**) linear speed of FAGV, (**b**) angular velocity of FAGV.

Figure 9, Figure 10 and Figure 11 show the path planning of the algorithm under the original evaluation function and the improved evaluation function, respectively. The path starting point is S, and the target point is T. The linear velocity of the initial state of the FAGV is v = 0 m/s, and the initial angular velocity is ω = 0 m/s.

Comparing the original path and the improved path in Figure 9, it can be seen that after the introduction of the new evaluation function, the local path of FAGV is closer to the global path under the premise of ensuring safe obstacle avoidance. After improving the DWA, the actual moving distance of FAGV is significantly reduced, and the overall path smoothness is better, which conforms to the dynamic characteristics of FAGV. Comparing the linear velocity of FAGV in Figure 10a and Figure 11a, when approaching the obstacle and the target point, the speed of the FAGV controlled by the original evaluation function is larger, between 0.6 m/s and 0.7 m/s. After improving the evaluation function, the speed of FAGV approaching the obstacle is between 0.3 m/s and 0.4 m/s or 0.5 m/s and 0.6 m/s, and the speed is smaller when avoiding obstacles. Driving at high speed near obstacles cannot guarantee the safety of forklifts and cargo, and will also cause the actual moving direction of the angular velocity of FAGV to deviate from the global path and the actual moving distance to be larger. After introducing the new evaluation function, the FAGV line speed is lower, which is in line with the principle of slowing down near obstacles. At the same time, comparing the area of the graph surrounded by the linear velocity image and the x-axis, it can be seen that the area is smaller after the improved evaluation function, which also verifies that the actual moving distance of the improved FAGV is smaller. By comparing Figure 10b and Figure 11b, it can be concluded that under the condition of the original evaluation function, the amplitude of forklift angular velocity is [−0.4,0.4]. After the introduction of the new evaluation function, the amplitude of FAGV angular velocity is [−0.3,0.4]. The amplitude of FAGV angular velocity becomes smaller, which shows that when approaching obstacles, FAGV angle adjustment is stable, jitter is small, and obstacle avoidance is smooth.

### 6.2. Simulation Results of Local Obstacle Avoidance

To test the effectiveness of the algorithm in the face of sudden obstacles, unknown static and dynamic obstacles are added to the raster map.

FAGV continuously scans the surrounding environment information when driving along the global shortest path planned by the improved A* algorithm, updates the rolling window, and determines whether there are unknown static or dynamic obstacles.

**Figure 12:**
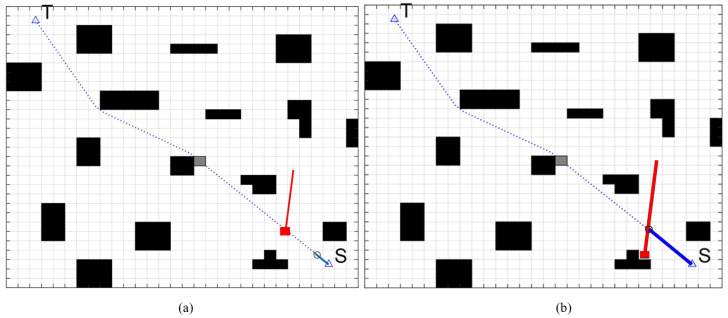
Dynamic obstacles for high-speed movement. (**a**) There is an unknown high-speed dynamic obstacle in front of the FAGV and it is predicted that the FAGV will not collide with the obstacle. (**b**) FAGV continues on the current path.

**Figure 13:**
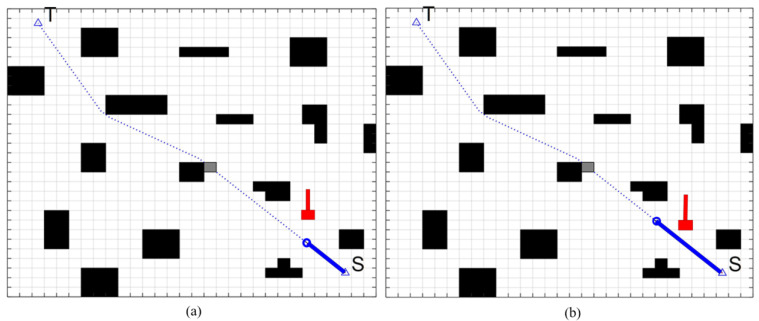
Dynamic obstacles for low-speed movement. (**a**) There is an unknown low -speed dynamic obstacle in front of the FAGV and it is predicted that the FAGV will not collide with the obstacle. (**b**) FAGV continues on the current path. When FAGV travels to the intersection point of the track, the obstacle is still not reached.

As shown in Figure 12 and Figure 13, the red block is an unknown dynamic obstacle, and the red line is an unknown dynamic obstacle motion route. FAGV continuously scans the surrounding environment information when driving along the global shortest path planned by the improved A* algorithm, updates the rolling window, and determines whether there are unknown static or dynamic obstacles. If an obstacle with too large or too small of a speed is detected and it is judged that the obstacle will not collide with FAGV, FAGV will continue to move forward along the global path.

**Figure 14:**
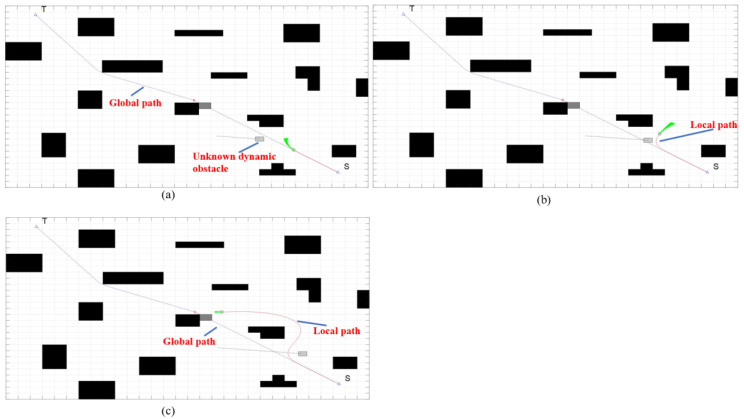
Dynamic path planning algorithm for FAGV returns a global path after avoiding dynamic obstacles. (**a**) FAGV detects a dynamic obstacle that is about to collide. (**b**) FAGV drives along the local path for obstacle avoidance. (**c**) FAGV returns the global path after obstacle avoidance.

Figure 14 shows that FAGV encounters unknown dynamic obstacles that will collide during driving. Figure 14a indicates that after the safety laser scanner and motion sensor detect the unknown dynamic obstacles, the rolling window method predicts that the FAGV is about to collide with the obstacle on the global path. According to the obstacle avoidance strategy, the forklift calls DWA for local path planning. Figure 14b indicates that the FAGV travels along the local path, and there is no collision when the dynamic obstacle travels to the original path of the forklift. Figure 14c indicates that after the FAGV avoids obstacles, it returns to the global path to continue driving.

**Figure 15:**
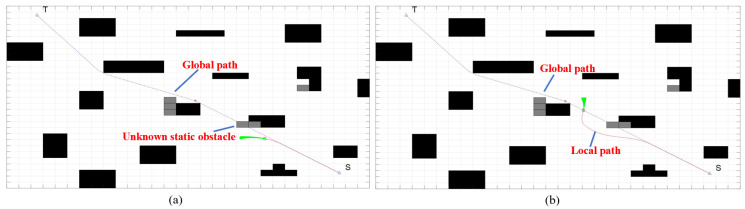
Dynamic path planning algorithm for FAGV returns a global path after avoiding static obstacles. (**a**) FAGV detects a static obstacle that is about to collide. (**b**) FAGV returns to the global path after avoiding static obstacles.

As shown in Figure 15, when the safety laser scanner and motion sensor detect unknown static obstacles in the front path, the improved DWA is called for local path planning to avoid. After avoiding the unknown dynamic obstacles, the FAGV returns to the global path and continues to move toward the target point.

According to the above simulation, the dynamic path planning algorithm of FAGV based on A* and DWA proposed in this paper can find the shortest barrier-free path for FAGV and guide FAGV to avoid static and dynamic obstacles in a warehouse and complete the handling task safely and reliably.

Table 2 compares the five algorithms mentioned in this paper. It can be seen from the table that the hybrid algorithm proposed in this paper takes into account both global optimality and local optimality, and can avoid obstacles in a dynamic environment.

## 7. Conclusions

As heavy equipment in the storage environment, FAGVs need a simple and smooth driving path, and at the same time, they should be able to avoid sudden obstacles in the driving process. In view of the above two requirements, this paper proposes a hybrid dynamic path planning algorithm suitable for FAGV. Firstly, we improve the A* algorithm to make the global path smoother. Then, we design a new evaluation function to improve the global optimality and local optimality of DWA in path planning and combine the rolling window method to solve the dynamic obstacle avoidance problem of FAGV. The simulation results show that the number of path turns of the improved A* algorithm is reduced by 62.5%, the turning angle is smaller, and the smoothness is higher. The local path of the improved DWA planning is closer to the global optimal path, and the FAGV has lower linear velocity and smaller angular velocity amplitude when it is close to the obstacle, which conforms to the principle of slowing down near the obstacle. After the collision is predicted by the rolling window method, FAGV runs according to the local path to avoid obstacles and returns to the global optimal path in time after successful avoidance.

The algorithm proposed in this paper can be used for the path planning of the FAGV in the working environment. However, the map environment and positioning information obtained by the navigation laser scanner used in the FAGV is limited to the two-dimensional level, and a comprehensive map environment cannot be established. Therefore, it can be considered to add binocular cameras or other sensors to the FAGV. The combination of visual positioning and laser positioning is used to fuse the collected data through corresponding algorithms to increase the accuracy of the map environment and positioning information.

## Figures and Tables

**Table 1:** Speed Parameters of DW.

Parameter Name	Numerical Value
maximum/ minimum line velocity	1/0 m/s
maximum/ minimum angular velocity	0.35/−0.35 rad/s
maximum/ minimum linear acceleration	0.2/0 m/s^2^
maximum/ minimum angular acceleration	0.9/−0.9 rad/s^2^
prediction time T	3 s
interval time ∆t	0.1 s

**Table 2:** Comparison of algorithms mentioned in this paper.

Algorithm	Global Optimality	Smooth Path	Local Optimality	Deceleration Obstacle Avoidance	Dynamic Obstacle Avoidance
Tradition A*	**√**	**×**	**×**	**×**	**×**
Improved A*	**√**	**√**	**×**	**×**	**×**
Tradition DWA	**×**	**×**	**√**	**×**	**×**
Improved DWA	**√**	**√**	**√**	**√**	**×**
Hybrid Algorithm	**√**	**√**	**√**	**√**	**√**
